# Supplementary material for: Similarity-Based Virtual Screening to Find Antituberculosis Agents Based on Novel Scaffolds: Design, Syntheses and Pharmacological Assays
Source: Int J Mol Sci. 2022 Dec 1;23(23):15057. doi: 10.3390/ijms232315057 (PMC9737236; doi:10.3390/ijms232315057)
Supplement: Supplementary file 1 [file ijms-23-15057-s001.zip › Supporting Information 1 Table S1. Structures of active compounds.pdf]

Table S1. Structures of the 32 compounds in the group of active substances.

Name	Structure
Amikacin	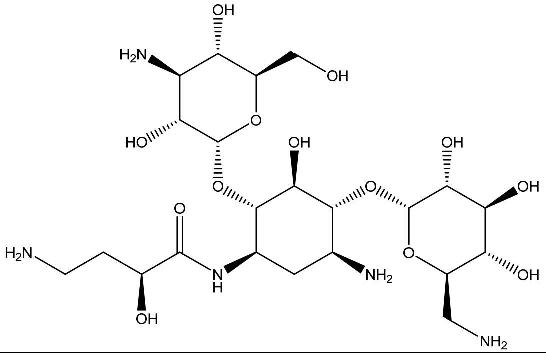
Azithromycin	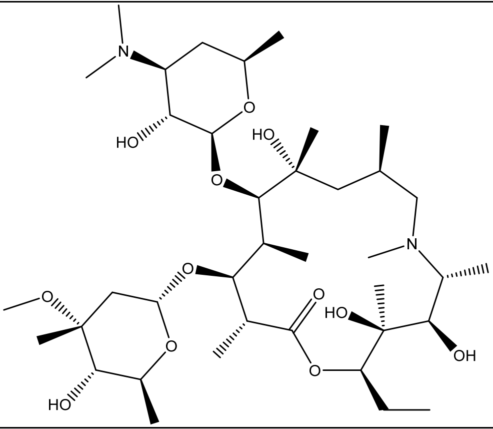
Capreomycin	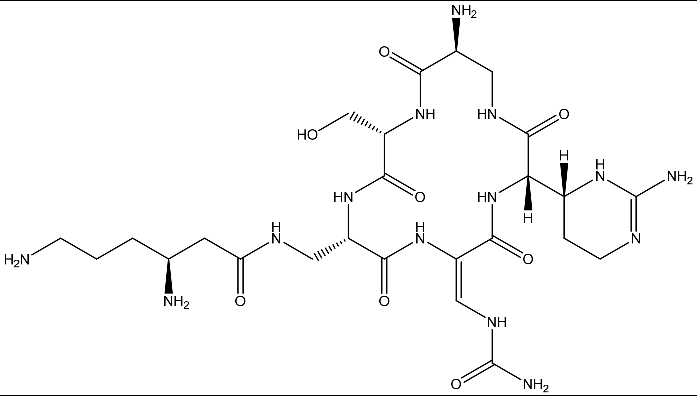
Ciprofloxacin	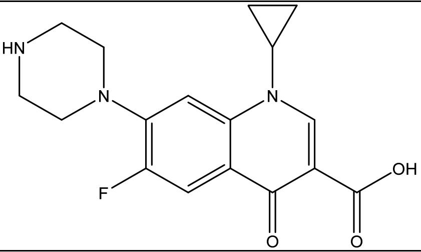

Clarithromycin	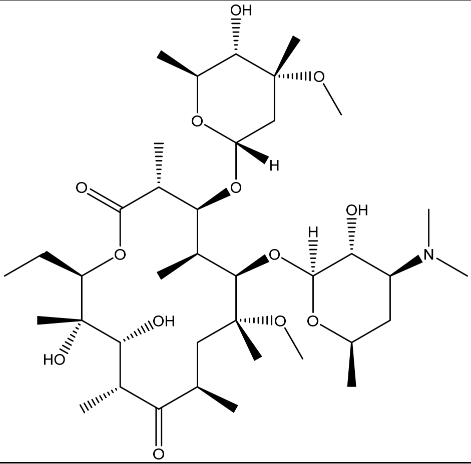
Clofazimine	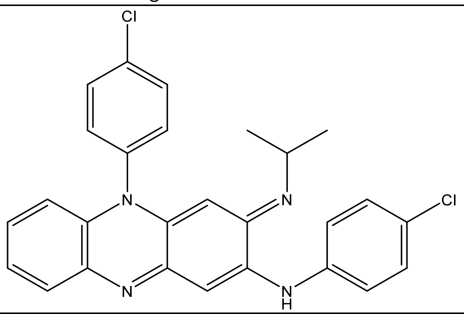
Dihydrostreptomycin	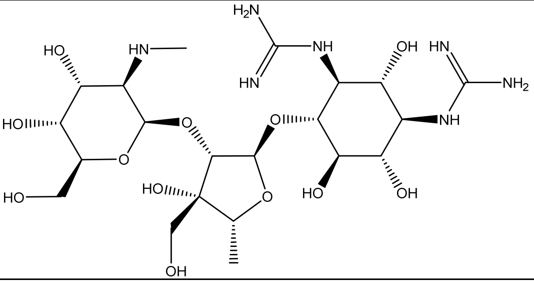
Enviomycin	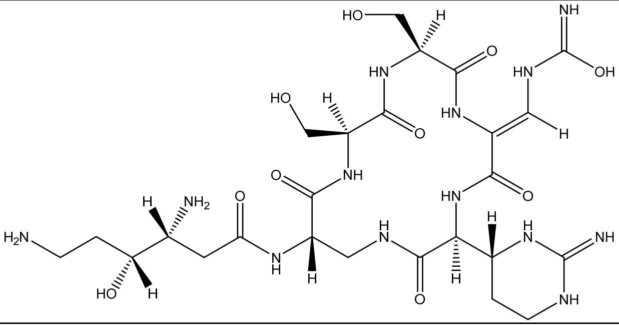
Ethambutol	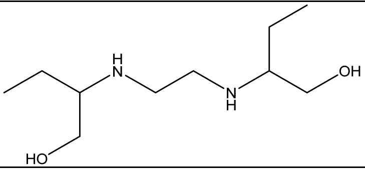
Ethionamide	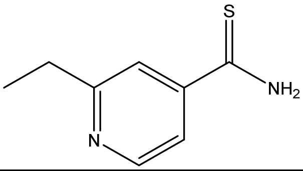

Gatifloxacin	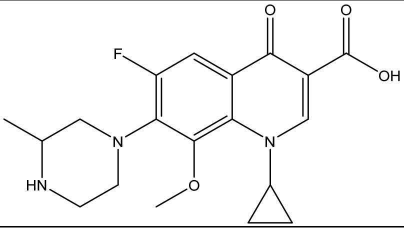
Imipenem	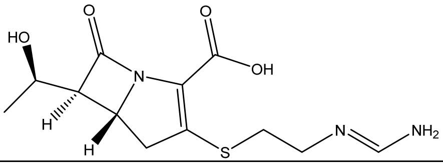
Isoniazid	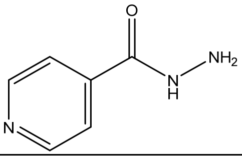
Kanamycin	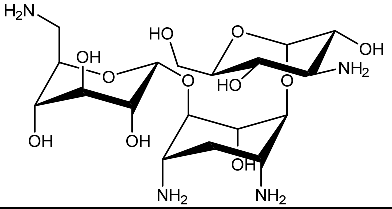
Morphazinamide	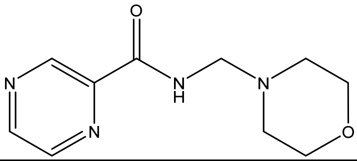
Moxifloxacin	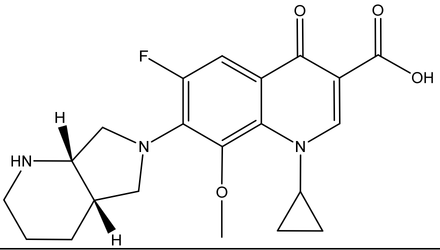
Neomycin	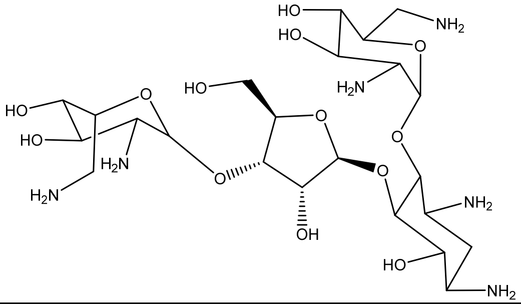
Ofloxacin	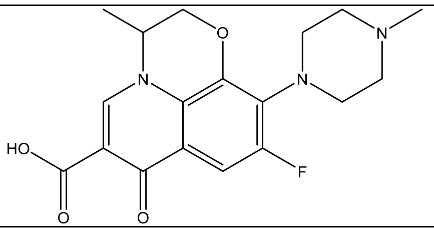
p-aminosalicylic acid	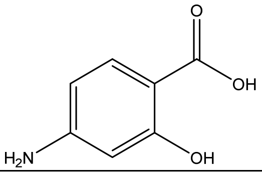

Phenyl P-aminosalicylate	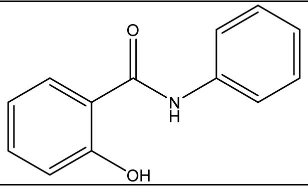
Pyrazinamide	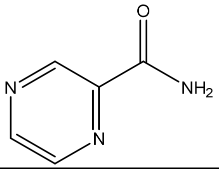
Rifabutin	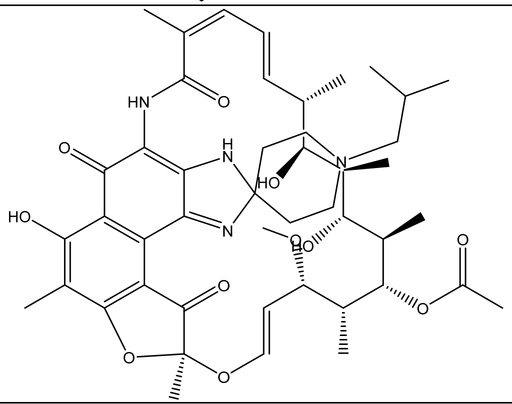
Rifampin	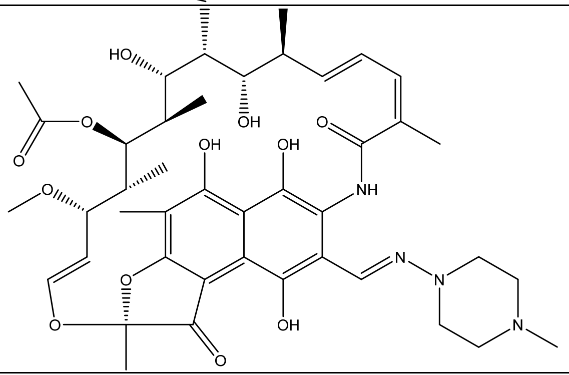
Salinazid	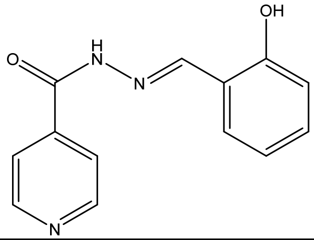
Sparfloxacin	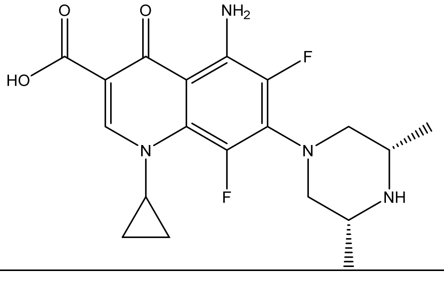

Streptomycin	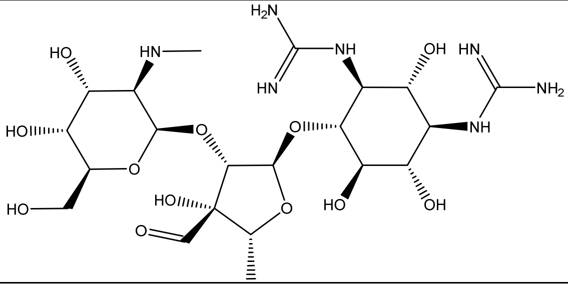
Streptonicozid	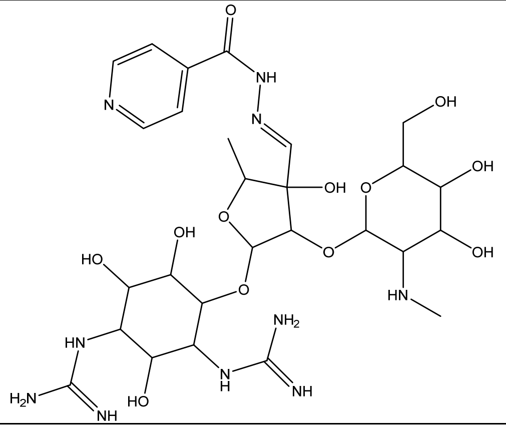
Tobramycin	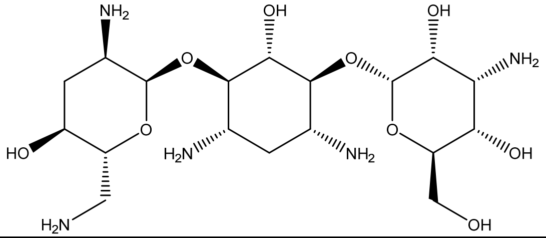
Tubercidin	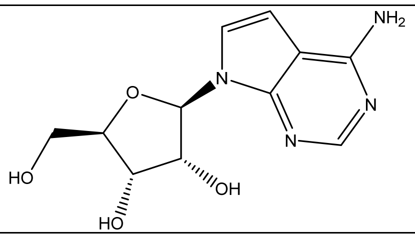
Tuberin	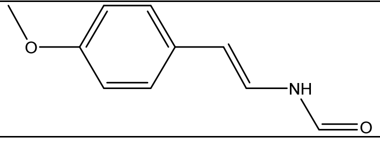
Verazide	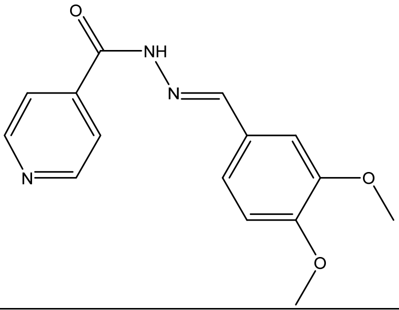

Viomycin	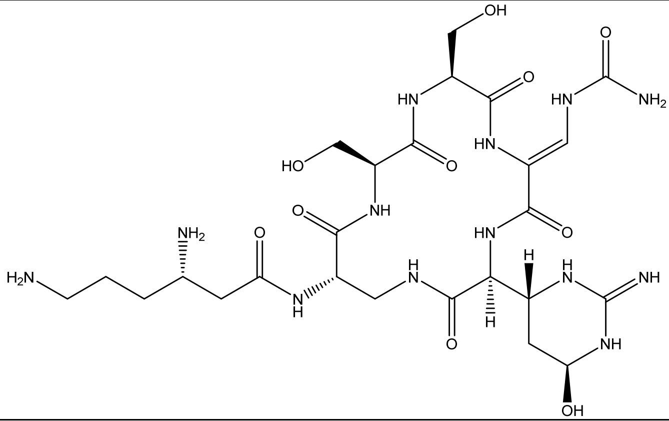 OH<br>O<br>HN<br>O<br>HN<br>NH2<br>HO<br>O<br>HN<br>O<br>NH<br>HN<br>O<br>NH2<br>O<br>H2N<br>N H<br>N H<br>H<br>H<br>N H<br>NH<br>NH<br>O<br>OH
