# Supplementary material for: Similarity-Based Virtual Screening to Find Antituberculosis Agents Based on Novel Scaffolds: Design, Syntheses and Pharmacological Assays
Source: Int J Mol Sci. 2022 Dec 1;23(23):15057. doi: 10.3390/ijms232315057 (PMC9737236; doi:10.3390/ijms232315057)
Supplement: Supplementary file 1 [file ijms-23-15057-s001.zip › Supporting Information 2 Table S2. Structures of inactive compounds.pdf]

Table S2. Structures of the 45 compounds in the group of inactive substances.

Name	Structure
Acifran	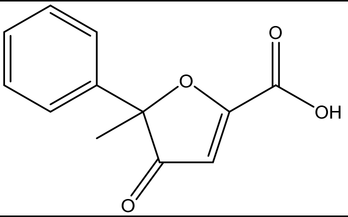
Acipimox	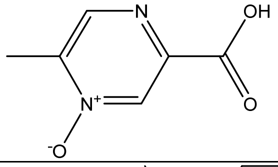
Acronine	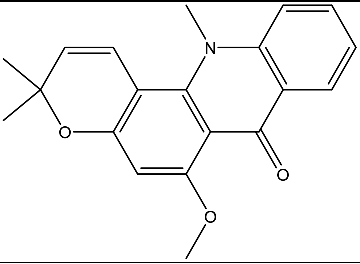
Aldicarb	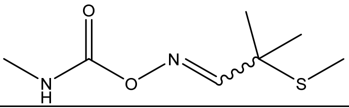
Allicin	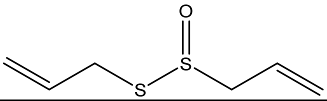
Alpidem	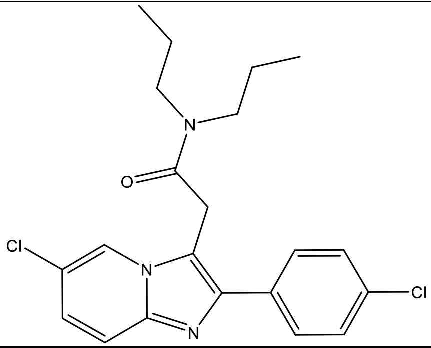
Alprazolam	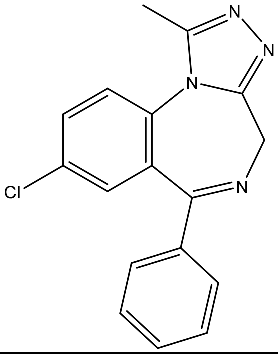

Altretamine	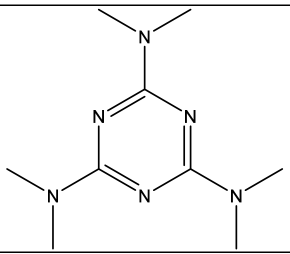
Aminopromazine	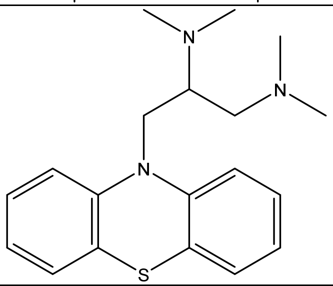
Aminothiazole	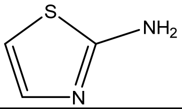
Amitraz	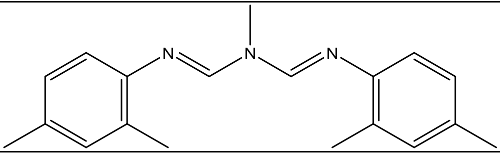
Amsacrine	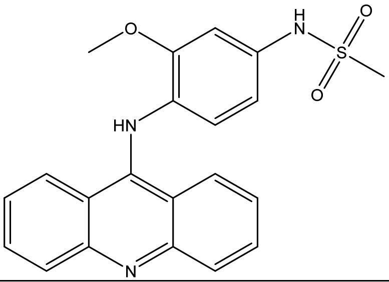
Antipyrine	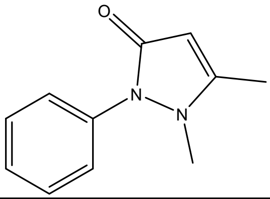
Antrafenine	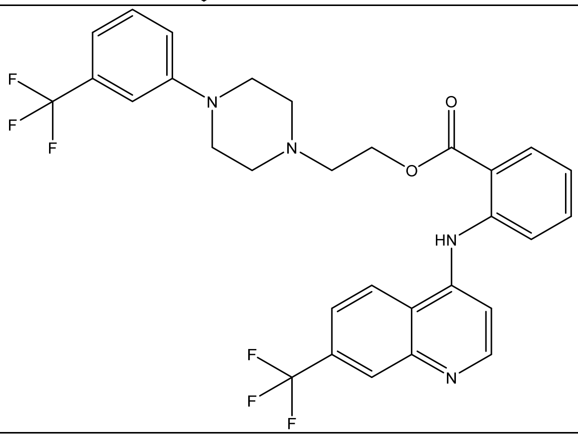

Azacosterol	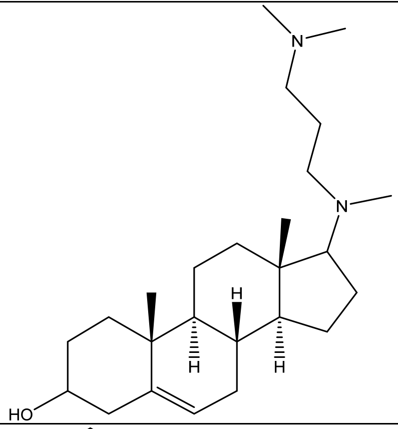
Azapicyl	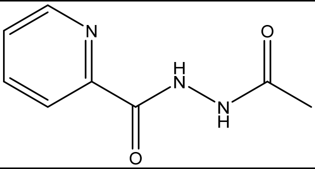
Azaserine	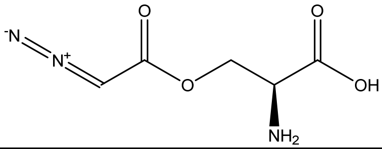
Beclobrate	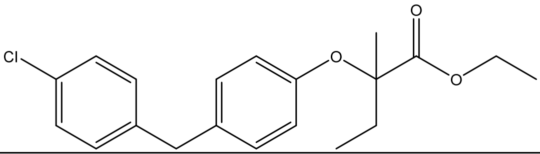
Benorylate	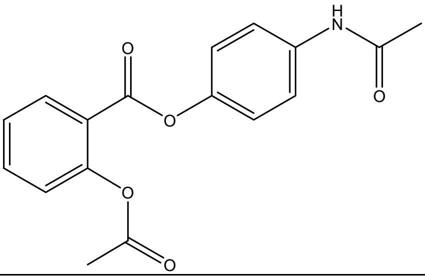
Benzoctamine	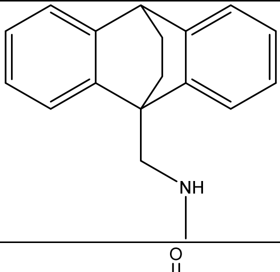
Benzoic Acid	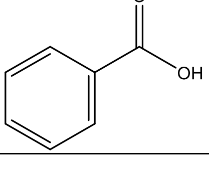

Bixin	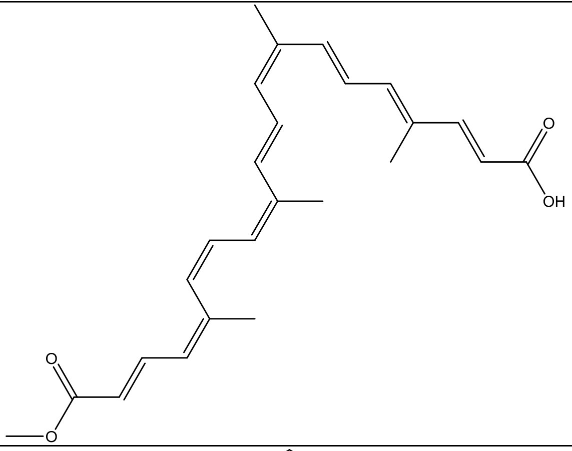
Brilliant Blue	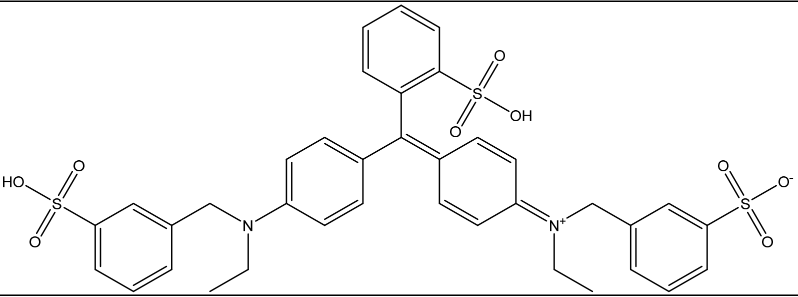
Bromazepam	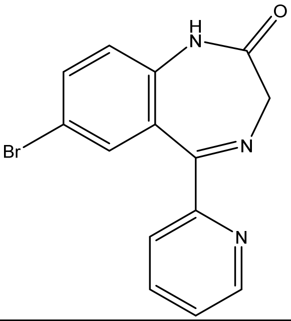
Buspirone	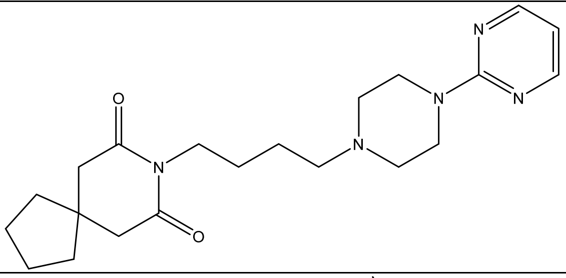
Butibufen	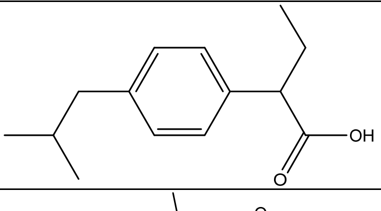
Camazepam	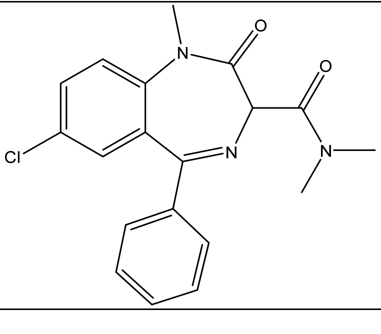

Canthaxanthin	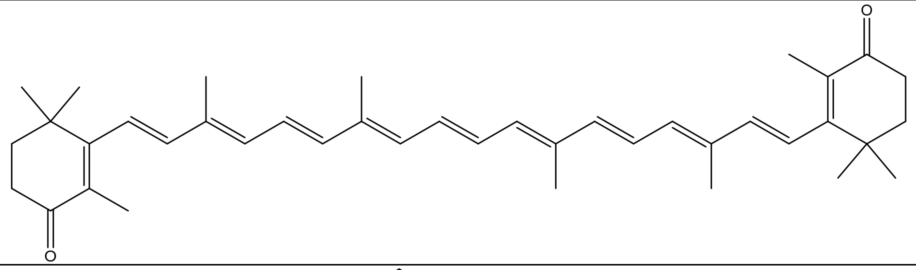
Captodiamine	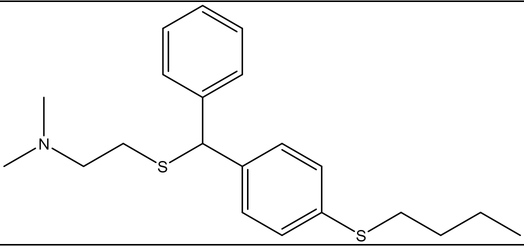
Carmofur	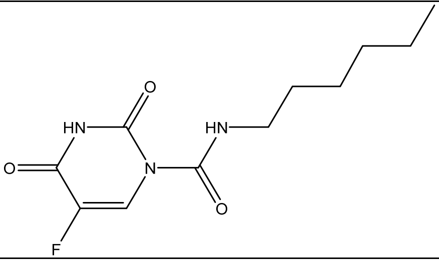
Carmustine	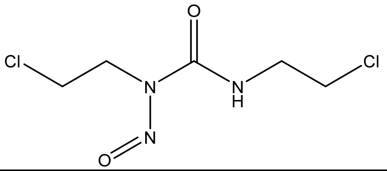
Carnitine	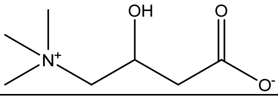
Carprofen	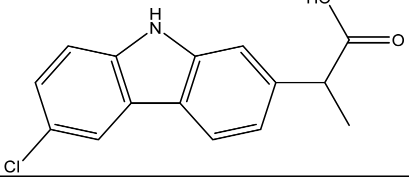
Chloropal	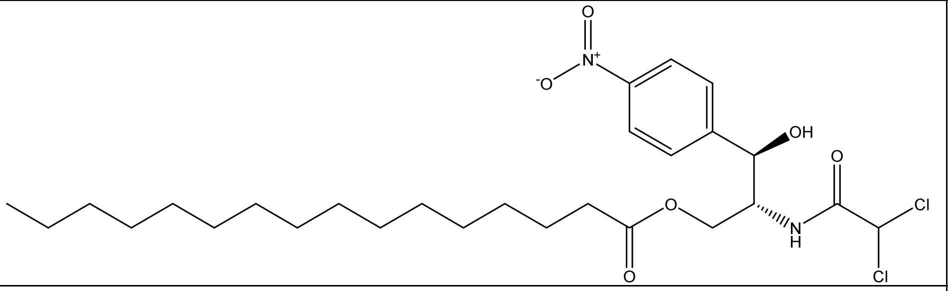
Clofibrate	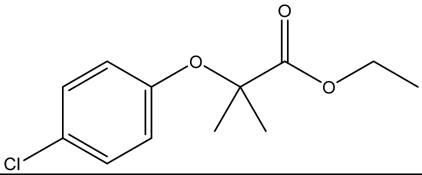

Dichlone	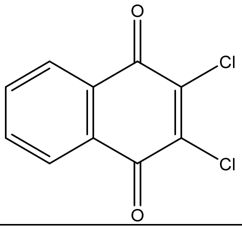
Etifoxin	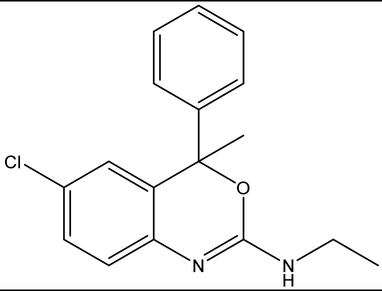
Feprazone	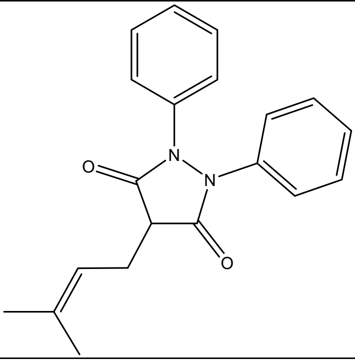
Genite	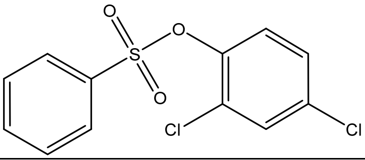
Glucosamine	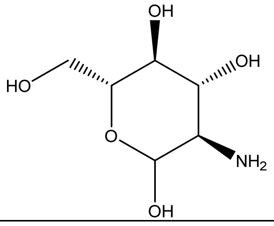
Ornithine	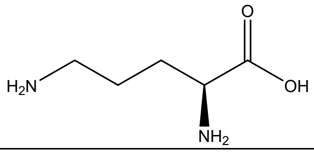
Paraoxon	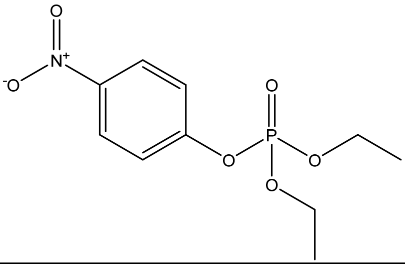

Piroxicam	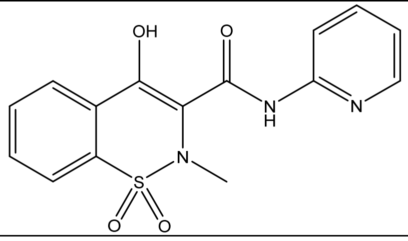
Prazepam	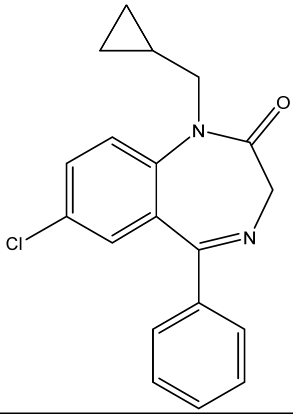
Theofibrate	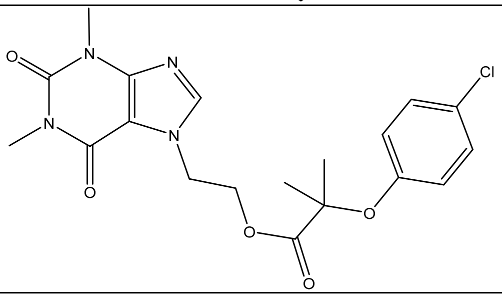
