# Supplementary material for: Similarity-Based Virtual Screening to Find Antituberculosis Agents Based on Novel Scaffolds: Design, Syntheses and Pharmacological Assays
Source: Int J Mol Sci. 2022 Dec 1;23(23):15057. doi: 10.3390/ijms232315057 (PMC9737236; doi:10.3390/ijms232315057)
Supplement: Supplementary file 1 [file ijms-23-15057-s001.zip › Supporting Information 3 Table S3. Threshods.pdf]

Supporting Information

Table S3. Descriptors and discriminant function with their corresponding thresholds.

		Thresholds				Thresholds	
Descriptor #	Parameter	Lower	Upper	Descriptor #	Parameter	Lower	Upper
1	N	7	49	47	^0D	0.19	7.64
2	V3	1	21	48	^1D	0.20	5.49
3	V4	0	10	49	^2D	0.25	6.76
4	R	1	18	50	^3D_P	0.18	7.13
5	W	45	8928	51	^3D_C	-0.01	1.83
6	L	3	20	52	^4D_P	0.14	6.32
7	PR0	0	1	53	^4D_C	0.00	0.22
8	PR1	0	13	54	$^4D_{PC}$	0.09	5.65
9	PR2	0	17	55	E_1	2.91	40.77
10	PR3	0	15	56	E_2	-4.52	9.16
11	^0X	4.70	33.67	57	E_3	-1.02	4.54
12	^1X	2.41	20.36	58	E_4	-0.93	2.36
13	^2X	1.48	18.41	59	E_5	-0.37	1.19
14	^3X_P	0.85	15.71	60	F_1	0.26	1.42
15	^3X_C	0.18	3.61	61	F_2	-0.16	0.33
16	^4X_P	0.27	12.25	62	F_3	-0.10	0.10
17	^4X_C	0.00	0.25	63	F_4	-0.05	0.05
18	$^4X_{PC}$	0.27	9.52	64	F_5	-0.03	0.03
19	$^0X^V$	3.64	26.02	65	^0C	1.01	1.34
20	$^1X^V$	1.67	14.87	66	^1C	1.04	1.51
21	$^2X^V$	0.90	11.65	67	^2C	1.08	1.77
22	$^3X_P^V$	0.40	8.58	68	^3C_P	1.10	2.37
23	$^3X_C^V$	0.07	1.81	69	^3C_C	0.98	2.87
24	$^4X_P^V$	0.12	5.92	70	^4C_P	1.15	3.07
25	$^4X_C^V$	0.00	0.05	71	^4C_C	0.00	9.13
26	$^4X_{PC}^V$	0.08	3.87	72	$^4C_{PC}$	1.17	3.41
27	G_1	1.75	17.25	73	^0Q	0.75	0.99
28	G_2	1.11	22.56	74	^1Q	0.66	0.96
29	G_3	0.63	6.75	75	^2Q	0.56	0.92
30	G_4	0.00	4.73	76	^3Q_P	0.42	0.91
31	G_5	0.00	2.96	77	^3Q_C	0.35	1.02
32	G_1^V	6.03	56.03	78	^4Q_P	0.33	0.87
33	G_2^V	4.20	31.72	79	^4Q_C	0.00	0.45
34	G_3^V	0.86	10.90	80	$^4Q_{PC}$	0.29	0.85
35	G_4^V	0.00	6.41	81	CG_1	0.10	0.56
36	G_5^V	0.00	4.05	82	CG_2	0.26	1.62
37	J_1	0.15	0.42	83	CG_3	0.46	2.18
38	J_2	0.09	0.68	84	CG_4	0.00	1.71
39	J_3	0.05	0.20	85	CG_5	0.00	5.86
40	J_4	0.00	0.13	86	QG_1	1.77	10.49
41	J_5	0.00	0.06	87	QG_2	0.62	3.78
42	J_1^V	0.57	1.57	88	QG_3	0.46	2.19
43	J_2^V	0.19	0.90	89	QG_4	0.00	2.94
44	J_3^V	0.09	0.24	90	QG_5	0.00	4.34
45	J_4^V	0.00	0.15	-	DF	0.00	4.50
46	J_5^V	0.00	0.09				
