# Supplementary material for: Similarity-Based Virtual Screening to Find Antituberculosis Agents Based on Novel Scaffolds: Design, Syntheses and Pharmacological Assays
Source: Int J Mol Sci. 2022 Dec 1;23(23):15057. doi: 10.3390/ijms232315057 (PMC9737236; doi:10.3390/ijms232315057)
Supplement: Supplementary file 1 [file ijms-23-15057-s001.zip › Supporting Information 4 Table S4. Intercorrelations.pdf]

Table S4. Intercorrelations between the variables of DF for the group of active compounds.

	J_1	J_1^v	J_3^v	^1D	^4C_c
J_1	1	-0.3959151	0.19469083	0.12647627	0.06154102
J_1^v	-0.3959151	1	0.42390454	0.13859051	-0.27254996
J_3^v	0.19469083	0.42390454	1	0.36881239	0.09421997
^1D	0.12647627	0.13859051	0.36881239	1	0.15407574
^4C_c	0.06154102	-0.27254996	0.09421997	0.15407574	1
