# Supplementary material for: Similarity-Based Virtual Screening to Find Antituberculosis Agents Based on Novel Scaffolds: Design, Syntheses and Pharmacological Assays
Source: Int J Mol Sci. 2022 Dec 1;23(23):15057. doi: 10.3390/ijms232315057 (PMC9737236; doi:10.3390/ijms232315057)
Supplement: Supplementary file 1 [file ijms-23-15057-s001.zip › Supporting Information 6 Spectra.pdf]

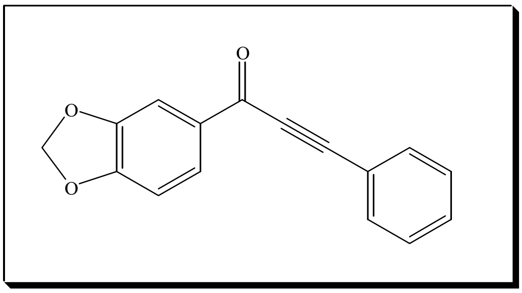

4a

IR (film)

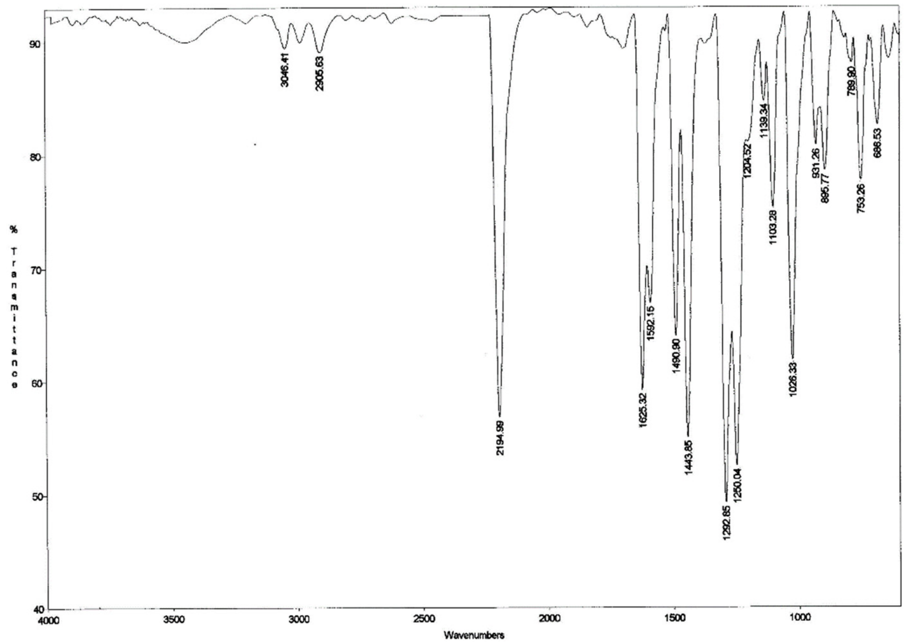

^{1}H-RMN (200 MHz, $CDCl_3$)

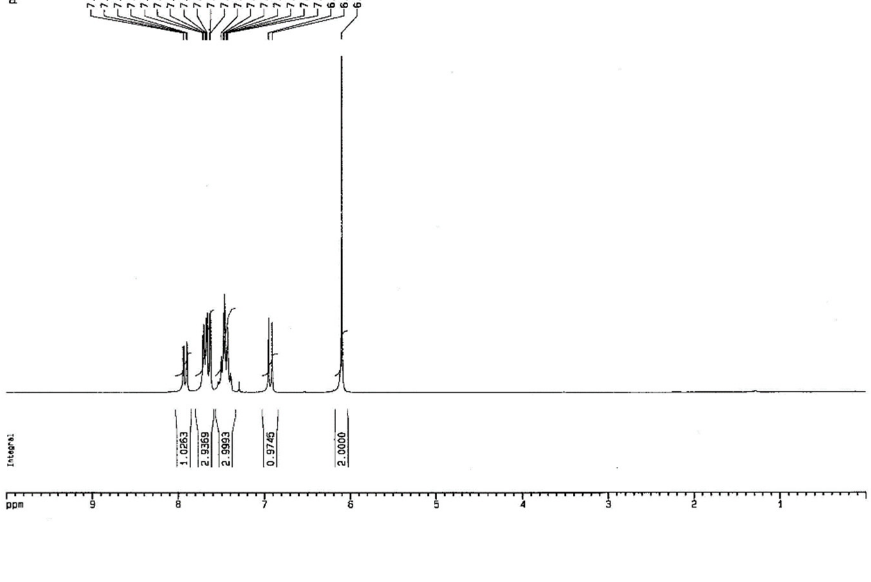

^{13}**C-RMN** (50 MHz, $CDCl_3$)

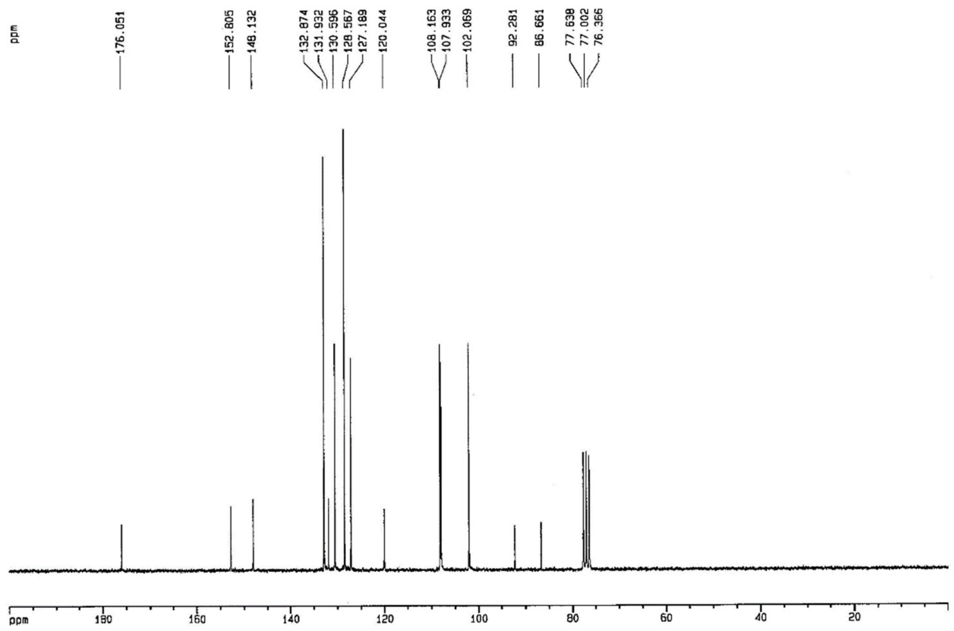

Figure S1. IR and NMR spectra of the compound **4a**.

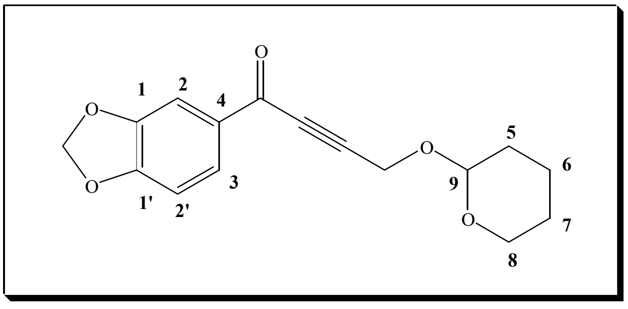

4b

IR (film)

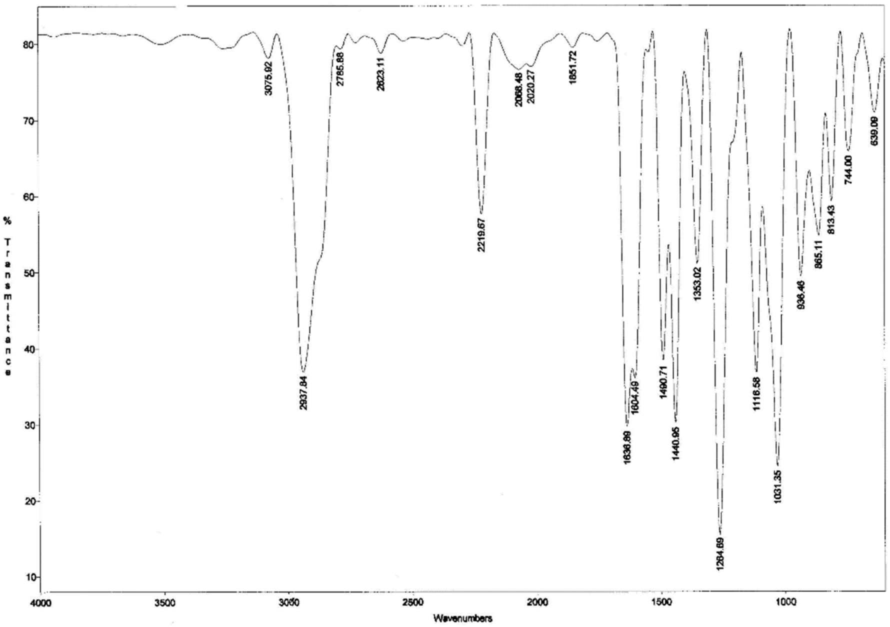

^{1}H-RMN (200 MHz, $CDCl_3$)

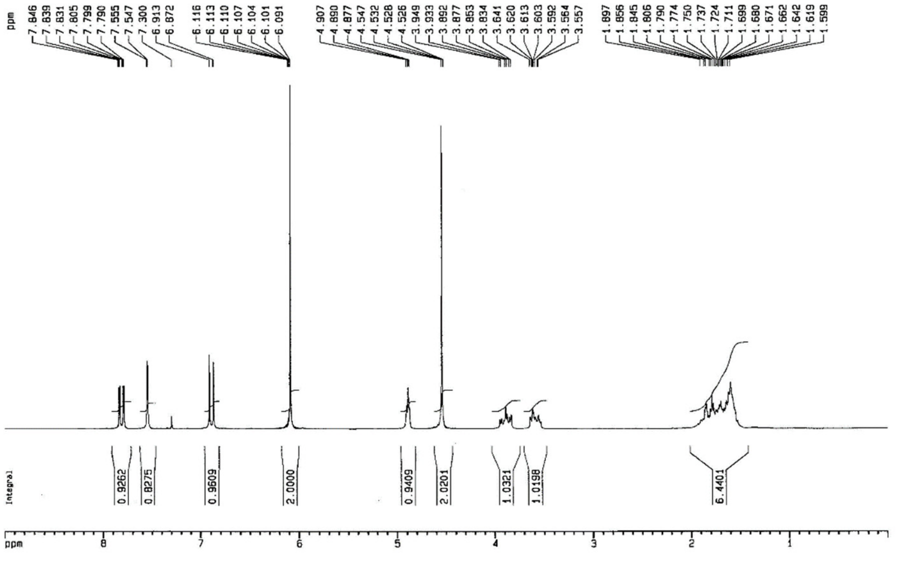

^{13}C-RMN (50 MHz, $CDCl_3$)

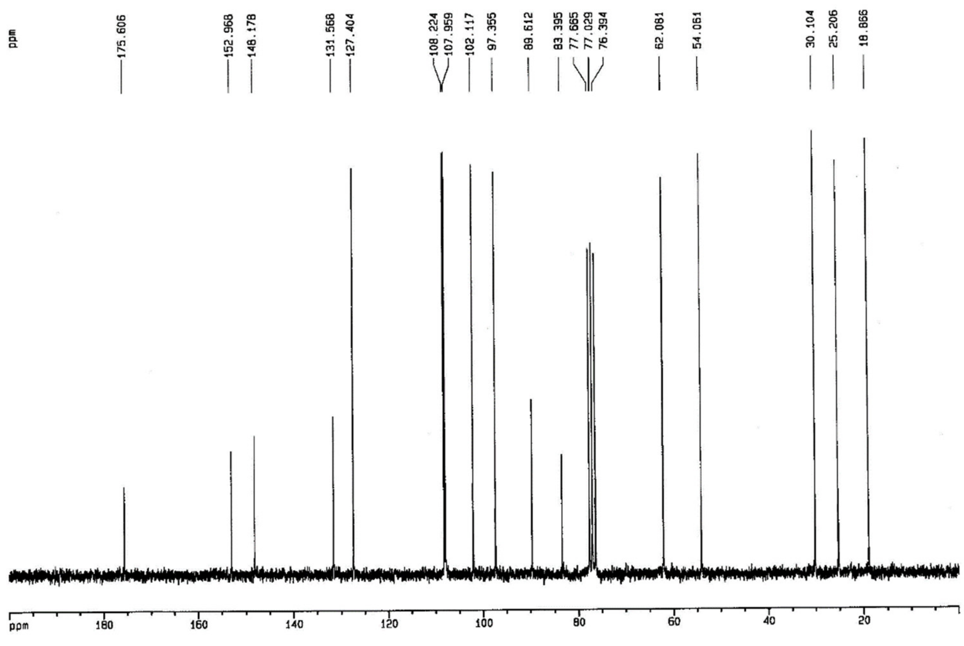

Figure S2. IR and NMR spectra of the compound **4b**.

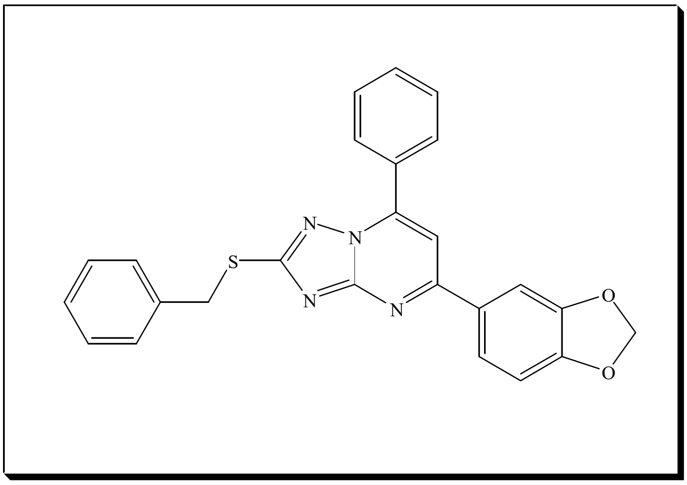

6a

IR (KBr)

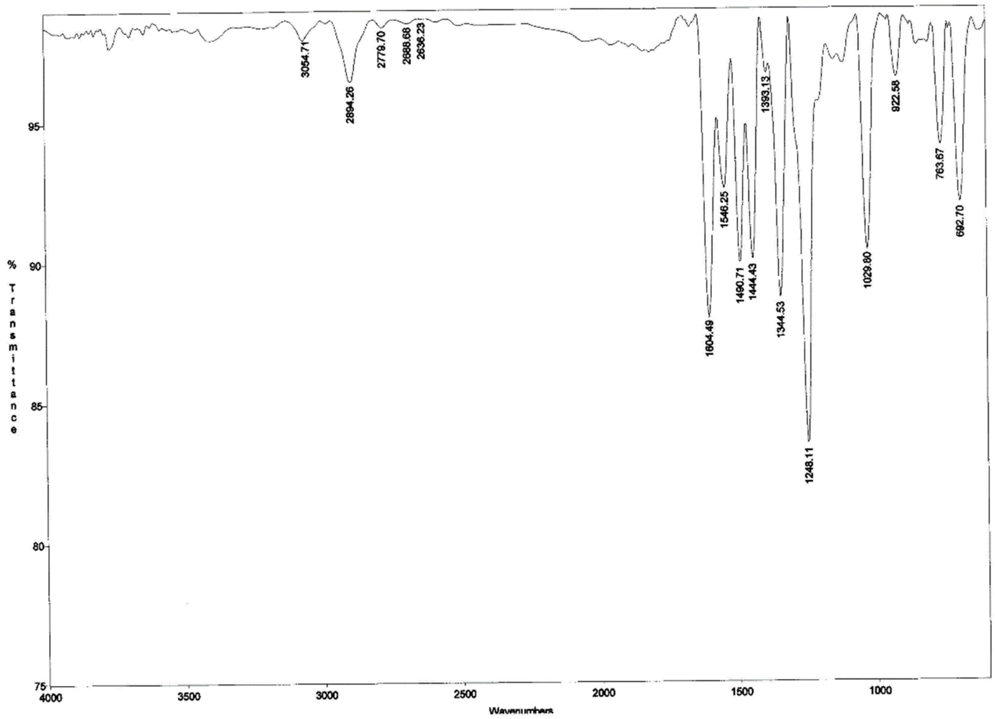

[1]**H-RMN** (200 MHz, DMSO-d6)

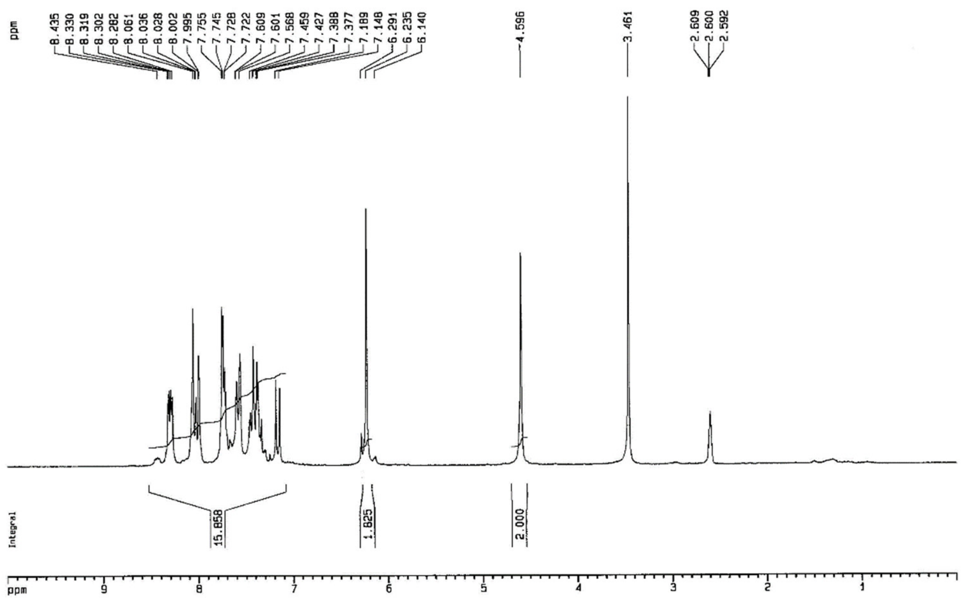

^{13}C-RMN (50 MHz, DMSO-d6)

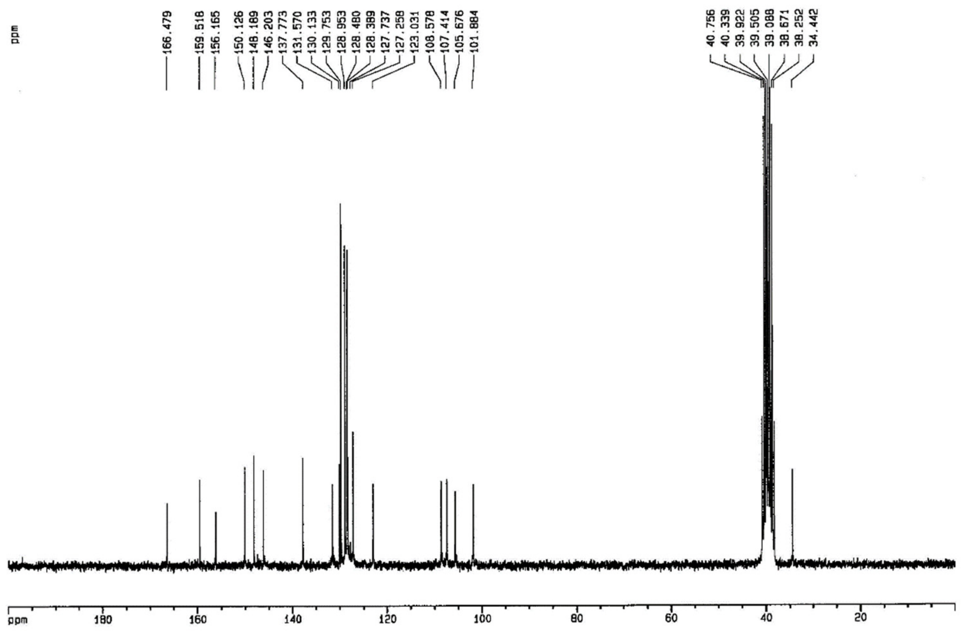

Figure S3. IR and NMR spectra of the compound **6a**.
